# Glycerol monolaurate inhibits *Francisella novicida* growth and is produced intracellularly in an ISG15-dependent manner

**DOI:** 10.17912/micropub.biology.000905

**Published:** 2023-10-27

**Authors:** Ellen M. Upton, Patrick M. Schlievert, Yifeng Zhang, Adam J. Rauckhorst, Eric B. Taylor, Lilliana Radoshevich

**Affiliations:** 1 Department of Microbiology and Immunology, University of Iowa, Carver College of Medicine, Iowa City, Iowa, USA; 2 Department of Molecular Physiology and Biophysics, University of Iowa, Carver College of Medicine, Iowa City, Iowa, USA; 3 Fraternal Order of Eagles Diabetes Research Center Metabolomics Core Facility, University of Iowa, Carver College of Medicine, Iowa City, Iowa, USA

## Abstract

Glycerol Monolaurate (GML) is a naturally occurring fatty acid monoester with antimicrobial properties. *Francisella tularensis* is an agent of bioterrorism known for its unique lipopolysaccharide structure and low immunogenicity. Here we assessed whether exogenous GML would inhibit the growth of *Francisella novicida* . GML potently impeded *Francisella* growth and survival *in vitro* . To appraise the metabolic response to infection, we used GC-MS to survey the metabolome, and surprisingly, observed intracellular GML production following *Francisella* infection. Notably, the ubiquitin-like protein ISG15 was necessary for increased GML levels induced by bacterial infection, and enhanced ISG15 conjugation correlated with GML levels following serum starvation.

**Figure 1. Glycerol monolaurate (GML) inhibits Francisella novicida growth and can be produced intracellularly following infection or serum starvation:**
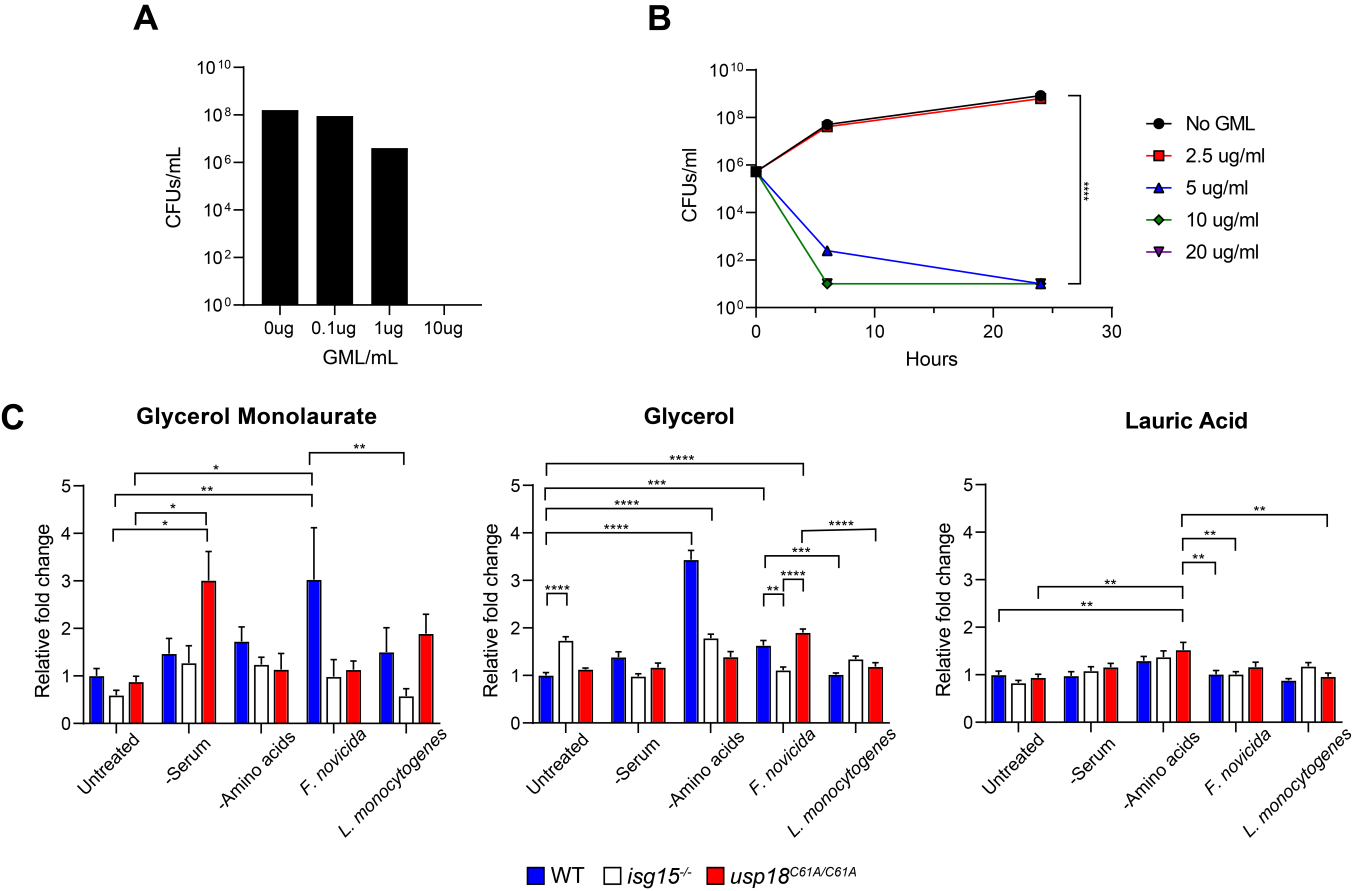
(A) *F. novicida* growth was assessed at 48 hours post-inoculation with the indicated concentrations of GML (B) *F. novicida* growth with varying concentrations of GML as assessed over a time course of 24 hours, with significant inhibition of growth observed at 5 μg/ml of GML, as indicated by a ≥3 log reduction in colony-forming units/ml compared to no-GML control. The mean of triplicate data is reported. (C) Metabolites were measured in Mouse Embryonic Fibroblasts (MEFs) with absent ISG15 ( *isg15 ^-/-^* ) or enhanced ISGylation ( *usp18 ^C61A/C61A^* ) following the indicated treatment; 2 hours of amino acid starvation and 24 hours of all other treatments. Glycerol monolaurate and its precursors are shown; n=6 biological replicates per group. Statistical significance was assessed via two-way ANOVA and Tukey’s multiple comparisons, adjusted p-values are shown; * ≤ p=0.05, ** ≤ p=0.005, *** ≤ p=0.0005, **** ≤ p=0.0001.

## Description

Glycerol monolaurate (GML) is a fatty acid monoester that has antimicrobial effects against a variety of human pathogens, and at the same time prevents harmful inflammation on body surfaces (Li et al., 2009; Lin, 2009; Peterson & Schlievert, 2006; Projan et al., 1994; Schlievert et al., 2018; Schlievert & Peterson, 2012; Strandberg, 2010) . GML is found readily in nature in coconut and palm oil. It is generally recognized as a safe compound by the Food And Drug Administration and is used as an emulsifier and preservative in many food and cosmetic products (Fosdick et al., 2021; Hess et al., 2014; Luo et al., 2022; Zhang et al., 2016) . GML is not routinely found in the human body, except it is present at high concentrations in breast milk where it exhibits both antibacterial and anti-inflammatory activity (Schlievert et al., 2019) . The lauric acid side chain of GML is optimal for its antibacterial properties since fatty acid monoesters with longer or shorter side chain length lose activity (Kabara, 1981; Kabara, 1984; Schlievert & Peterson, 2012; Yoon et al., 2018) ; lauric acid is just long enough to span one-half of the lipid bilayer. GML antibacterial activity appears to result from the dissipation of the potential difference across the bacterial plasma membrane (Schlievert & Peterson, 2012) . The bactericidal activity of GML is strongly correlated to lipopolysaccharide (LPS) makeup, as *Enterobacteriaceae* are not susceptible to GML with a fully intact LPS layer (Schlievert & Peterson, 2012) . However, disruption of LPS or human pathogens without an intact LPS layer, for example, *Neisseria* , *Haemophilus* , and *Gardnerella,* are susceptible to killing by GML (Schlievert & Peterson, 2012) .

Our study sought to assess the role of GML in antibacterial activity against *Francisella novicida* , a subspecies of the category A select agent of bioterrorism *Francisella tularensis* (Kingry & Petersen, 2014) . *Francisella* has a unique LPS composition with four acyl groups making up the Lipid A molecule instead of the six that are present in *E. coli* LPS (Dueñas et al., 2006; Okan & Kasper, 2013; Vinogradov et al., 2002). It also lacks the two phosphate groups usually observed on the hydrophobic anchor of Lipid A and instead has two hydroxyl groups (Okan & Kasper, 2013) . These modifications result in evasion of Toll-Like Receptor 4 (TLR4) stimulation and allow *Francisella* to replicate undetected by the immune system (Dueñas et al., 2006; Hajjar et al., 2006). *F. novicida* is a zoonotic pathogen that is genetically similar to *F. tularensis* and is often used as a Biosafety Level 2 model of *Francisella* infection due to its genetic tractability and safety for humans. *F. novicida* retains the unique Lipid A structure characteristic of *F. tularensis* but harbors slight differences in the O-antigen and LPS core (Gunn & Ernst, 2007; Kingry & Petersen, 2014) . Although *F. novicida* is not pathogenic to humans, it is highly virulent in mice. The organism stimulates Interleukin 12 (IL-12) and Tumor Necrosis Factor Alpha (TNF-α) production from mouse splenocytes at a greater level than other *Francisella* subspecies such as *F. tularensis* Live Vaccine Strain (LVS), yet both strains have dramatically reduced immune activation compared to that induced by the LPS of *E. coli* (Dreisbach et al., 2000; Kieffer et al., 2003) .

Since the bactericidal activity of GML is dependent on LPS structure in Gram-negative bacteria we hypothesized that the modifications on the LPS of *F. novicida* could increase susceptibility to GML. Indeed, following a 48-hour incubation with varying concentrations of GML we saw reduced growth starting at 1 μg/mL of GML and no growth at 10 μg/mL ( Figure 1A ). We subsequently performed a time course assay and assessed the growth of *F. novicida* at 6- and 24-hours post-inoculation with varying concentrations of GML. Here we observed a reduction in growth at 5 μg/mL GML as early as 6 hours ( Figure 1B ). However, incubation with 2.5 μg/ml of GML was insufficient to reduce *F. novicida* growth ( Figure 1B ). Altogether these data support our hypothesis that GML could prevent the growth of *F. novicida* in liquid culture. Additionally, GML was bactericidal for *F. novicida* at GML concentrations in the same range as required to kill other Gram-negative bacteria lacking an intact *Enterobacteriaceae* LPS.

*Francisella* is primarily an intracellular pathogen replicating in macrophages and alveolar epithelial cells *in vivo* (Celli & Zahrt, 2013; Craven et al., 2008; Hall et al., 2007; Steiner et al., 2017) . Thus, we hypothesized that GML could potentially be produced intracellularly, noting however, that up to this time, human breast milk is the only known source of GML produced in the human body. To address this hypothesis, we assessed levels of GML and its precursors, glycerol and lauric acid, via GC-MS in Mouse Embryonic Fibroblasts (MEFs) following either serum or amino acid starvation, or infection with either *F. novicida* or a Gram-positive intracellular pathogen, *L. monocytogenes* (Radoshevich & Cossart, 2018) *.* We did not observe any biologically relevant significant changes to lauric acid during any of the conditions tested. However, we detected accumulation of glycerol following amino-acid deprivation in wild-type cells, though not following bacterial infection. Most notably, we observed increased GML in wild-type MEFs following *F. novicida* infection ( Figure 1C ). This indicated that epithelial cells can produce GML in response to infection or cell stress, which was previously unknown. In comparison, we observed no increase in GML production following intracellular *L. monocytogenes* infection, even though GML has been shown as an antibacterial agent against the pathogen (Wang & Johnson, 1992; Yoon et al., 2018) . This difference could be attributed to the unique method of hijacking host glucose, carbon sources, and lipids by each pathogen (Eisenreich et al., 2010; Grubmüller et al., 2014; Meibom & Charbit, 2010; Ziveri et al., 2017).

We conducted the initial metabolomic study to assess the role of the interferon-induced ubiquitin-like protein, ISG15 (Perng et al., 2018) , on cellular metabolism. Our previous work suggests that ISG15 is antibacterial (Radoshevich et al., 2015) and can modify enzymes which are critical to metabolic pathways (Zhang et al., 2019) . To our surprise, the presence of ISG15 was necessary for the production of GML in MEFs, since deletion abrogated the increase in GML following *F. novicida* infection ( Figure 1C ). Because the cells were harvested in bulk, GML could be produced by the invading bacteria. In cells with enhanced ISGylation ( *usp18 ^C61A/C61A^* ), induced by catalytic inactivation of the ISG15-specific protease USP18 (Ketscher et al., 2015) , we observed an accumulation of GML following serum starvation in cells ( Figure 1C ). This notable result indicated that unchecked covalent modification by ISG15 could lead to an intracellular accumulation of GML following infection or starvation.

ISG15 is emerging as a potent regulator of metabolism, especially in the liver and in adipose tissue. Infection with *L. monocytogenes* provokes ISG15 modification at active sites and dimerization domains of metabolic enzymes, which could block their function (Zhang et al., 2019) . *Isg15 ^-/-^* mice have basal decreases in glycogen stores and decreased oxygen consumption following Coxsackie virus infection (Kespohl et al., 2020) . ISG15 modification of glycolytic enzymes in adipocytes also reduces lactate levels and increases thermogenesis, which protects from high fat diet induced obesity (Yan et al., 2021) . In addition, ISG15 regulates lipid metabolism in bone-marrow-derived macrophages, which manifests as a reduction of neutral lipids in its absence (Albert et al., 2022) . Our data indicate that GML can be generated intracellularly and that ISG15 regulates its production. Future work will determine how this occurs and whether it is a direct or indirect mechanism.

Finally, our studies demonstrate for the first time that breast milk is not the only mammalian source of GML. Our findings lay the groundwork for investigating the mechanistic consequences of GML secretion or retention in the cytosol. ISG15 conjugation has been shown to restrict exosome secretion (Villarroya-Beltri et al., 2016) , which could potentially lead to the cytosolic accumulation of GML. GML from human breast milk directly targets the membrane integrity of extracellular bacteria (Schlievert & Peterson, 2012) . By contrast, we posit that intracellular GML may be packaged into toxic lipid droplets to target cytosolic pathogens (Bosch et al., 2020) . Interestingly, ISG15 mediates the oligomerization of RNF213, a giant E3 ligase known to stabilize lipid droplets and dock on the surface of intracellular pathogens (Sugihara et al., 2019; Thery et al., 2021) . We hypothesize that this could result in the targeted trafficking of GML to bacterial surfaces thus avoiding damage to host membranes. Overall, this study opens a new avenue for the assessment of GML as an effector of intracellular defense mechanisms alongside its role as a natural oral or topical antibiotic.

## Methods

*Bacterial culture*

Stock cultures of *F. novicida* were prepared after growth to stationary phase in Todd Hewitt (Difco Laboratories, Detroit, MI). For growth in the presence and absence of GML, *F. novicida* was inoculated into new Todd Hewitt broths with initial inoculums of 10 ^6^ /ml. Growth was measured by viable plate counts on Todd Hewitt agar after culturing at 37 °C with 200 revolutions per minute shaking for indicated times. GML is soluble in aqueous solutions at concentrations up to 100 µg/ml. Thus, aqueous solutions of GML (0 to 20 µg/ml) in Todd Hewitt broths were prepared for testing of antimicrobial activity. No-GML controls were prepared similarly except GML was omitted.

*Cell Culture metabolomic sample preparation*

MEFs were seeded in standard tissue culture dishes coated with 20 µg/mL human fibronectin (Corning, 354008). For amino acid starvation, cells were washed once with PBS and fed with HBSS, calcium, and magnesium, with no phenol red (Gibco, 14025092) for 2 h. For serum starvation, cells were washed once with 1x DPBS and fed with serum-free DMEM for 24 h. For *Listeria monocytogenes* infection, overnight culture of *L. monocytogenes* strain EGD was diluted in Brain Heart Infusion media (BD) and grown to exponential phase (OD 0.8–1), washed three times and resuspended in serum-free DMEM at MOI of 10. A fixed volume was then added to each well. Cells were centrifuged for 1 min at 201 × g to synchronize infection. The cells were then incubated with the bacteria for 1 h at 37 °C, 5% CO _2_ . Following this incubation, the cells were washed with 1× DPBS and then fed with full growth medium (with 10% fetal bovine serum) supplemented with 20 μg/ml gentamicin to kill extracellular bacteria for an additional 23 h. For *Francisella novicida* infection, overnight culture of *F. novicida* was diluted in BHI, pH 6.5 (BD), grown to exponential phase (OD 0.5), washed three times in serum-free DMEM, and resuspended in serum-free DMEM at MOI of 100. A fixed volume was then added to each well. Cells were centrifuged for 1 min at 201 × g to synchronize infection. The cells were then incubated with the bacteria for 1 h at 37 °C, 5% CO _2_ . Following this incubation, the cells were washed at room temperature with 1× DPBS, and then fed with full growth medium (with 10% fetal bovine serum) supplemented with 20 μg/ml gentamicin to kill extracellular bacteria for an additional 23 h. Untreated control cells, amino acid-starved, serum-starved, *L. monocytogenes-* infected, and *F. novicida-* infected cells were immediately placed on ice at the indicated time, washed twice with ice-cold PBS followed by two washes with ice-cold miliQ-H _2_ O. Cell monolayers were snap frozen in gaseous phase liquid nitrogen after all liquids were removed from the cells. Cells were then wrapped in parafilm and immediately transferred to the -80 °C freezer for storage upon flash freezing.

*GC-MS metabolomic analysis*

Snap frozen cell monolayers were lyophilized before being scraped into 1 ml of ice-cold 2:2:1 acetonitrile: methanol: water containing a mixture of labeled internal standards including citric acid (2,2,4,4-D4), succinic acid (2,2,3,3-D4), l-valine (2,3,4,4,4,5,5,5-D8), l-glutamic acid ( ^13^ C5), l-glutamine ( ^13^ C5), l-lysine ( ^13^ C6), l-methionine ( ^13^ C5), and l-tryptophan ( ^13^ C11) obtained from Cambridge Isotope Laboratories, Inc. The resulting mixture was transferred to a microcentrifuge tube and snap-frozen in liquid nitrogen. Frozen extraction mixtures were thawed with bath sonication, vortexed for 10 minutes, and rotated at -20 °C for 60 minutes. Next, crude homogenates were centrifuged at 21,000 x *g* for 10 minutes at 4 °C, and the resulting cleared metabolite extracts were collected. 150 µL of the metabolite extract or a pooled quality control (QC) sample prepared by mixing equal volumes from each sample were transferred to glass autosampler vials and dried to completeness using a SpeedVac vacuum concentrator.

Samples were prepared for GC-MS analysis by derivatization with methoxyamine (MOX) + N-methyl-N-(trimethylsilyl) trifluoroacetamide (MSTFA). First, dried extracts were resuspended in 30 μL of pyridine containing 11.4 mg/mL of MOX. Samples were vortexed for 10 min and heated at 60 °C for 60 min. Next, 20 uL of MSTFA was added to the pyridine/MOX derivatized samples. Samples were vortexed for 5 min and heated at 60 °C for 30 min.

1 μL of sample was injected into a Trace 1300 GC (Thermo) operated in split mode (split ratio: 20:1; split flow: 24 μL/min, purge flow: 5 mL/min, Carrier mode: Constant Flow, Carrier flow rate: 1.2 mL/min). Separation was accomplished using a standard fused silica TraceGold TG-5SilMS column (Thermo). The temperature gradient was: 80 °C for 3 min, ramped at a rate of 20 °C/min to 280 °C and held for 8 min. Ions were detected using an ISQ-LT single quadrupole mass spectrometer operated from 3.90 to 21.00 min in EI mode (−70eV) using select ion monitoring (SIM). The pooled QC sample was analyzed at the beginning and at the end of the GC/MS run, as well as about every eight injections throughout. Between sample runs, the injection syringe was washed 3 times with methanol and 3 times with pyridine. The mass spectrometer was tuned and calibrated daily.

Acquired GC–MS data were processed using the Thermo Scientific TraceFinder (4.1 and 5.1) software. Targeted metabolites were identified based on the University of Iowa Metabolomics Core facility standard-confirmed, in-house library defining a target ion and at least 1 confirming ion and retention time. The NOREVA tool used the QC sample analyzed throughout the instrument run to apply local polynomial fits to metabolite peak areas and correct for instrument drift. NOREVA corrected data were normalized to the D4-succinate signal/sample to control for extraction, derivatization (GC), and/or loading effects.

## Reagents

**Table:** 

Food grade GML	Colonial Chemical Company, South Pittsburg, TN

**Bacterial strains:**

**Table:** 

*Francisella novicida* strain U112	available upon request
*Listeria monocytogenes* strain EGD	available upon request
